# Correlation functions for Elekta aSi EPIDs used as transit dosimeter for open fields

**DOI:** 10.1120/jacmp.v12i1.3279

**Published:** 2010-10-27

**Authors:** Savino Cilla, Andrea Fidanzio, Francesca Greco, Domenico Sabatino, Aniello Russo, Laura Gargiulo, Luigi Azario, Angelo Piermattei

**Affiliations:** ^1^ U.O di Fisica Sanitaria, Centro di Ricerca e Formazione ad Alta Tecnologia nelle Scienze Biomediche Università Cattolica del Sacro Cuore Campobasso Italy; ^2^ Istituto di Fisica Università Cattolica del Sacro Cuore Roma Italy; ^3^ U.O. di Radioterapia Casa di cura Marco Polo USI Roma Italy

**Keywords:** *in‐vivo* dosimetry, EPID calibration, quality assurance, transit dosimetry

## Abstract

*In‐vivo* dosimetry techniques are currently being applied only by a few Centers because they require time‐consuming implementation measurements, and workload for detector positioning and data analysis. The transit *in‐vivo* dosimetry performed by the electronic portal imaging device (EPID) avoids the problem of solid‐state detector positioning on the patient. Moreover, the dosimetric characterization of the recent Elekta aSi EPIDs in terms of signal stability and linearity make these detectors useful for the transit *in‐vivo* dosimetry with 6, 10 and 15 MV photon beams. However, the implementation of the EPID transit dosimetry requires several measurements. Recently, the present authors have developed an *in‐vivo* dosimetry method for 3D CRT based on correlation functions defined by the ratios between the transit signal, $s_{t}(w,L)$, by the EPID and the phantom midplane dose, $D_{m}(w,L)$, at the source to axis distance (SAD) as a function of the phantom thickness, w, and the square field dimensions, L. When the phantom midplane was positioned at distance, d, from the SAD, the ratios $s_{t}(w,L)/s_{t}^{'}(d,w,L)$ were used to take into account the variation of the scattered photon contributions on the EPID as a function of d and L.

The aim of this paper is the implementation of a procedure that uses generalized correlation functions obtained by nine Elekta Precise linac beams. The procedure can be used by other Elekta Precise linacs equipped with the same aSi EPIDs, assuming the stabilities of the beam output factors and the EPID signals. The procedure here reported avoids measurements in solid water equivalent phantoms needed to implement the *in‐vivo* dosimetry method in the radiotherapy department. A tolerance level ranging between ±5% and ±6% (depending on the type of tumor) was estimated for the comparison between the reconstructed isocenter dose, $D_{\text{iso}}$, and the computed dose, $D_{\text{iso},\text{TPS}}$, by the treatment planning system (TPS).

PACS number: 87.55.Qr; 87.56.Fc

## I. INTRODUCTION

*In‐vivo* dosimetry, by monitoring the actual dose received by the patient during treatment, is the ultimate check of a quality assurance program. In this procedure, the dose reconstructions performed during treatment are compared to an expected dose supplied by the treatment planning system (TPS). Discrepancies may be due to possible errors from previous steps in the radiotherapy process such as errors in the data transfer from the TPS to the radiotherapy unit, errors in the functioning of the treatment equipment, and errors in the accuracy of the dose calculation algorithms employed by the TPS, in addition to errors due to the patient setup or patient morphology changes.

A standard *in‐vivo* dosimetry technique is based on the entrance dose reconstruction using a solid‐state detector on the patient surface.^(^ ^1^ ^)^ These *in‐vivo* dosimetry techniques are generally applied only for an initial check because they require workload for detector positioning and corrections for their X‐ray fluence absorption.

The increasing complexity of techniques in radiotherapy requires an accurate verification of the dose delivered to the patient, and several studies have addressed reconstruction of dose delivered to the patient during the treatment by means of electronic portal imaging devices (EPIDs).^(^ ^2^ ^)^ When compared to the traditional EPIDs such as fluoroscopic screen/camera‐based and liquid‐filled matrix ionization chambers, the new generation of EPIDs, equipped with amorphous silicon (aSi) flat panels, supply more stable transit signals and are suitable as transit detectors. Recently, the authors have developed an *in‐vivo* dosimetry method based on correlation functions defined by the ratios between the transit signal, $s_{t}$, measured by aSi EPIDs, and the solid water phantom midplane doses, $D_{m}$, measured by an ion chamber positioned along the central axis.^(^ ^3^ ^)^

The aim of the present paper is the determination of the generalized correlation functions for the recent Elekta IviewGT aSi EPIDs needed to implement an *in‐vivo* transit dosimetry method for the 3D conformed radiotherapy (3DCRT). Use of these correlation functions avoids the need for measurements in solid water phantom.

## II. MATERIALS AND METHODS

### A. Linac equipments

Table 1 reports some characteristics of the linacs examined in this work which are used at the Università Cattolica del Sacro Cuore (UCSC) of Campobasso and at the Unione Sanitaria Internazionale (USI) of Rome. In particular, nine X‐ray beams of 6, 10 and 15 MV supplied by three Elekta Precise linacs (Elekta, Stockholm, Sweden) have been used to obtain the generalized correlation functions for the implementation of an *in‐vivo* dosimetry method. The linacs were equipped with EPIDs IviewGT Elekta, based on the aSi panel XRD 1640 AL5 (PerkinElmer Optoelectronics, Fremont, CA USA). The sensitive layer is based on aSi sensors operating as a two‐dimensional photodiode array. The sensitive layer consists of 1024×1024 pixels with a pitch of 400 μm, resulting in an active area of $409.6\times 409.6\;{\text{mm}}^{2}$. Back‐projected at the source– axis distance (SAD), this corresponds to an area of $259\times 259\;{\text{mm}}^{2}$ and a pitch of 253 μm. A more detailed description of the functionality and basic properties of such devices is reported in the literature.^(^ ^4^ ^,^ ^5^ ^)^ Above the detector, a copper plate with a thickness of 1 mm, acts as build‐up material, and the copper plate source–EPID distance (SED) is fixed around 159 cm (Table 1). However, the EPID can be in a retracted position when its use is not required. The Elekta Precise linacs were equipped with a multileaf collimator (MLC) that consists of two opposed banks carrying 40 leafs each with a 1 cm width at the isocenter. The X‐ray beams were calibrated following the IAEA 398 protocol^(^ ^6^ ^)^ using a $10\times 10\;{\text{cm}}^{2}$ field size at the source–phantom distance (SSD) equal to 100 cm, coincident with the SAD. At the reference depth, $z_{\text{ref}}$, equal to 10 cm in water phantom, the reference dose was equal to 1 cGy/MU for the UCSC beams, while the beams at the USI were calibrated with 1cGy/MU at the depth of maximum dose, $d_{\text{max}}$.

**Table 1:** Source EPID distances, SED, and the index quality ${\text{TPR}}_{20,10}$ for the three linac used in this work.

	*SED (cm)*	*6 MV*	*10 MV*	*15 MV*
Linac A (UCSC)	159.0	0.683	0.730	0.759
Linac B (UCSC)	159.5	0.683	0.731	0.759
Linac C (USI)	158.2	0.686	0.736	0.760

The quality index of each beam was obtained by the tissue phantom ratios ${\text{TPR}}_{20,10}$,^(^ ^6^ ^)^ (hereafter named TPR), measured as the ratio between the doses at the water depth of 20 cm and 10 cm, respectively, with an accuracy of 0.3% (Table 1).

### B. Transit dosimetry implementation

The method here proposed for the *in‐vivo* dose reconstruction at the isocenter is based on a set of measurements of: (i) dose values by a cylindrical ionchamber PTW, model TM31010 (PTW Freiburg, Germany) positioned at the SAD along the beam central axis coincident with the midplane of a solid water‐equivalent phantom, (SWEP) (Gammex Middleton, WS), and (ii) the transit signal by the EPID below the SWEP at the SED, measured on the beam central axis. The measurements have been carried out using phantom thicknesses w=10,22,30 and 42 cm, and square field sides L=4,8,10,12,16 and 20 cm defined at the SAD. Each measurement was obtained with 100 MU supplied with the clinical monitor unit rate 400MU/min, which was used at the UCSC, and 200 MU/min used at the USI.

**Figure 1:**
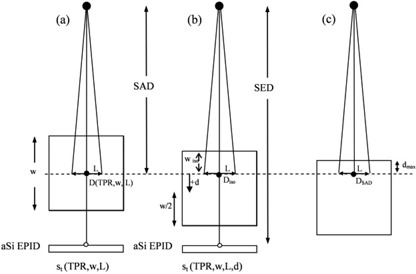
Setup used to measure the phantom midplane doses, D(TPR,w/2,L), and the EPID transit signals $s_{t}(\text{TPR},w,L)$ and $S_{t}(\text{TPR},w,L,d)$ where w is the phantom thickness, L is the side of the square field and the SED is the source to EPID distance: a) reference configuration with the phantom midplane at the source to axis distance SAD=100 cm. The ion chamber was positioned at the phantom midplane (•) to determine D(TPR,w,L), while $s_{t}(\text{TPR},w,L)$ was measured in the point (◯) on the beam central axis; b) the phantom midplane is at the distance, d, below the SAD. The dose, $D_{\text{iso}}$, at the isocenter point can be obtained by Eq. (3); c) phantom setup used to determine the $D_{\text{SAD}}$ at the depth, $d_{\text{max}}$, of the maximum dose for a $10\times 10\;{\text{cm}}^{2}$ field.

Figure 1(a) shows the experimental setup used to determine for every TPR, the midplane doses per MU, D(TPR,w,L), and the transit signals per MU, $s_{t}(\text{TPR},w,L)$. Figure 1(b) shows an experimental setup used to measure the transit signals per MU, $S_{t}(\text{TPR},w,L,d)$, when the phantom midplane was shifted a distance, d, from the SAD. These last values were used to determine the empirical factors f(TPR,d,L)

(1) $$f(\text{TPR},d,L)=s_{t}(\text{TPR},w,L)/s_{t}(\text{TPR},w,L,d)$$

that took into account the variations of the scattered photon contributions on the EPID due to the different midplane phantom positions as respect to the SAD. In previous papers,^(^ ^7^ ^,^ ^8^ ^)^ it was shown that, for distances, d, in the range of ±7 cm, the f(TPR,d,L) factors were independent of the thickness, w, within ±0.5%. Hence, we can report the factors in Eq. (1) as a function of d and L for every quality beam.

Defining the correlation function F(TPR,w,L) as the ratios

(2) $$F(\text{TPR},w,L)=\frac{s_{t}(\text{TPR},w,L)}{D(\text{TPR},w,L)}$$

the dose $D(\text{TPR}{,}_{\text{wiso}},L)$ at depth $w_{\text{iso}}$ can be determined (referring to Fig. 1) by

(3) $$D(\text{TPR},w_{\text{iso}},L)=S_{t}(\text{TPR},w,L,d)\cdot \left[\frac{f(\text{TPR},d,L)}{F(\text{TPR},w,L)}{\text{TMR}}_{w/2}^{w_{\text{iso}}}(L)\right]$$

where $S_{t}(\text{TPR},w,L,d)$ is the integral transit signal and the ${\text{TMR}}_{w/2}^{w_{\text{iso}}}(L)$ is the ratio between the Tissue Maximum Ratios evaluated at the depths, $w_{\text{iso}}$, and w/2, respectively. The accuracy of Eq. (3) was estimated equal to ±4.5% also in inhomogeneous phantoms where *w* was obtained in terms of radiological thickness.^(^ ^7^ ^,^ ^8^ ^)^

The correlation functions F(TPR,w,L) of Eq. (2) had to be determined for every linac beam because they depend on the beam MU calibration and on the Center's EPID sensitivity.

The aim of this work has been the determination of the generalized data for the ratios in Eq. (2) dependent, for every pair of (w, L), on the beam quality index TPR.

#### B.1 Midplane dose measurements

A water equivalency correction factor $k_{\text{WE}}$ for the SWEP was determined as the ratio between the chamber reading in natural water and that in solid phantom, at the same linear depth of 10 cm for the 6, 10 and 15 MV photon beams with a field $10\times 10\;{\text{cm}}^{2}$ in size. So the ionchamber reading in SWEP was multiplied for the factor, $k_{\text{WE}}$, before applying the IAEA code^(^ ^6^ ^)^ to obtain the dose to water.

The beam MU calibration can differ between the two Centers. To take this into account a factor, $k_{0}$, was defined as the ratio between two dose values at the depth, $d_{\text{max}}$, coincident with the SAD (Fig. 1(c) for a field $10\times 10\;{\text{cm}}^{2}$

(4) $$k_{0}=D_{\text{SAD}}^{0}/D_{\text{SAD}}$$

where $D_{\text{SAD}}^{0}=1\;\text{cGy}/\text{MU}$ and $D_{\text{SAD}}$ is the specific dose of the beam measured in our institution. Multiplying D(TPR,w,L) by $k_{0}$

(5) $$D^{0}(\text{TPR},w,L)=D(\text{TPR},w,L)\cdot k_{0}$$

by which a set of dose per MU values, $D^{0}(\text{TPR},w,L)$, in terms of cGy/MU, independent of the MU calibration adopted by the two Centers, were obtained. In other words, for the two types of data (w,L) the dose per MU obtained by Eq. (5) is dependent on the TPR index only.

#### B.2 Portal imaging measurements

An Elekta aSi EPID frame is defined as the raw signal s'(x,y) from one readout of the entire EPID panel, where x and y indicate the matrix pixel coordinates. Every frame is generated in 434 ms. The portal images are obtained by the integrated signals over the total beam‐on time multiplied by a pixel scaling factor, (PSF), to produce a quality image.^(^ ^9^ ^)^ The pixel signals are stored as a 16‐bit number and are inversely proportional to the dose. In this work, the portal images have been evaluated using an in‐house developed software in MATLAB version 7.1 (The Mathworks Inc., Nantick, Massachussets) environment. More specifically, after an irradiation on EPID, the raw pixel values s'(x,y) are normalized to give an optimized image for imaging purposes. This means that an image acquired with 10 MU has roughly the same grey levels as an image acquired with 100 MU (i.e., the same raw pixel values). The pixel scaling factor, PSF, represents the “scaling factor” of acquisition and it is supplied by the IviewGT software (version 3.3) at the end of the irradiation. The greater the dose, the smaller the PSF. In this manner, it is possible to obtain nonnormalized gray levels (i.e., the integrated pixel values s(x,y)) by subtracting the pixel values s'(x,y) from the number 65535 ($2^{16}-1$) and then dividing it by the PSF, according to equation:

(6) $$s(x,y)=\frac{65535-s'(x,y)}{\text{PSF}}$$

In particular, in this paper the EPID signal, s (at the SED), on the beam central axis, in terms of arbitrary units (au), was obtained by the average of the s(x,y) supplied by the 12×12 central pixels (an area of $4.8\times 4.8\;{\text{mm}}^{2}$) around the beam central axis.

The signals, s, were obtained for 6, 10 and 15 MV beams supplied by the three linacs, using a $10\times 10\;{\text{cm}}^{2}$ field at the SAD and delivering 100 MU each time. By doing so, the reproducibility of the EPID signals was checked twice a day, taking into account the daily linac output fluctuations. Moreover, the EPID dependence on the dose rate was investigated, changing the dose rate supplied by the linac (50, 100, 200 and 400 MU/min). To assess signal, s, linearity with MUs, the signal $s_{\text{MU}}$, obtained delivering 5, 20, 50, 100, 200, 400 and 1000 MUs, was used to determine the linearity correction factor, $k_{\text{lin}}$, defined as:

(7) $$k_{\text{lin}}=\frac{s}{s_{\text{MU}}}$$

where, s is the signal per MU obtained for 100 MUs.

Moreover, the ghosting effect that represents an artifact in the image due to signals present in frames subsequent to the frame in which it has been generated^(^ ^4^ ^)^ has been measured following the Van Esch approach.^(^ ^10^ ^)^

The aSi EPIDs operating in our Centers can supply different values of s for the same delivered dose and this yield can change in time. Moreover, through private communication of the Elekta, the fixed SEDs for different Precise linacs equipped with the aSi EPIDs ranged between 158.2 cm and 159.8 cm.

In this paper, a procedure that assures a stable calibration of the aSi EPIDs has been proposed. The first step was the determination of the $\bar{s}$ in terms of au/MU as the mean value of the s per MU obtained in the long‐term (six months) reproducibility checks for a $10\times 10\;{\text{cm}}^{2}$ field at the SAD and delivering 100 MUs. In a second step, the $\bar{s}$ signal was converted into centi‐CU per MU (cCU/MU), assuming that the $\bar{s}$ in au/MU was equal to 1 cCU/MU. This means that, at the SED, a sensitivity factor, $k_{s}$, in terms of cCU/au, was defined as

(8) $$k_{s}=\frac{1}{\bar{s}}$$

This way an integral signal, s, in terms of au (obtained by a number of MU) if multiplied by $k_{s}$ can be read in terms of cCU and this reading is independent of: (i) the EPID sensitivity, and (ii) the MU calibration of the megavoltage beam. In conclusion, aSi EPIDs with different sensitivities operating in different facilities at a fixed SED (approximately 159 cm) can supply, for every MU, the same $s\cdot k_{s}$ reading. Of course, if the signal, s, changes over a tolerance level from the $\bar{s}$, a new $k_{s}$ factor should be adopted to take into account the change of the EPID sensitivity.

The measured transit signals per MU, st(TPR,w,L), (obtained with the SWEP on the beam) were multiplied by $k_{s}$

(9) $$s_{t}^{0}(\text{TPR},w,L)=s_{t}(\text{TPR},w,L).k_{s}$$

obtaining generalized $s_{t}^{0}(\text{TPR},w,L)$ values, independent of both the MU calibration and the EPID sensitivity, but dependent on the TPR index only.

The measurements of the $S_{t}(\text{TPR},w,L,d)$ values were carried out positioning the phantom midplane below and above the SAD at distances, d, up to ±7 cm, as a function of w and L. These last data were used to determine the f(TPR,d,L) factor defined by Eq. (1).

### C. Reconstruction of the isocenter dose

A commercial software package (TableCurve 3D; SPSS‐Science, 2000) was used to find the surfaces of best fit to the measured data values for the generalized doses $D^{0}(\text{TPR},w,L)$ and the generalized transit signals $s_{t}^{0}(\text{TPR},w,L)$. TableCurve 3D is a linear and nonlinear surface‐fitting software package that automates the surface‐fitting process and, in a single processing step, instantly fits and ranks about a thousand equations, enabling users to find the ideal model to their 3D data within seconds. In this software, both linear and nonlinear equations can minimize the sum of squares of the residuals, where a residual is simply the difference between the experimental value and that computed from the surface–fit equation. The square of a residual is always positive, and thus reflects the magnitude of the residual.^(^ ^11^ ^)^

The generalized midplane doses $D^{0}(\text{TPR},w,L)$ (Eq. 5) were fitted by the surface equations

(10) $$D^{0}(\text{TPR},w,L)=a_{1}+a_{2}\;\text{TPR}+a_{3}\;w+a_{4}\;{\text{TPR}}^{2}+a_{5}\;w^{2}+a_{6}\;\text{TPR}\;w$$

where the six adjustable coefficients $a_{i}(I=1,\ldots ,6)$ are real numbers obtained through the fitting procedure.

The generalized transit signals $s_{t}^{{}^{\circ}}(\text{TPR},w,L)$ (Eq. 9) were fitted by the surface equations as

(11) $$\begin{array}{l} s_{t}^{0}(\text{TPR},w,L)=b_{1}+b_{2}\text{TPR}+b_{3}w+b_{4}{\text{TPR}}^{2}+b_{5}\;w^{2}+b_{6}\text{TPR}\;w+b_{7}{\text{TPR}}^{3}+b_{8}\;w^{3}+b_{9}\text{TPR}\\ w^{2}+b_{10}{\text{TPR}}^{2}w \end{array}$$

where the ten adjustable coefficients $b_{i}(I=1,\ldots ,10)$ are real numbers obtained through the fitting procedure.

Polynomial equations were chosen as a matter of simplicity. The number of adjustable parameters, 6 or 10, were chosen as the minimum that can provide residual values (i.e., the differences between the surface and experimental data) well within ±1.5%.

In clinical practice, the dose D(TPR, $w_{\text{iso}}$, L) in cGy for a beam quality index TPR (named $D_{\text{iso}}$ for simplicity) can be rewritten by Eq. (3) as

(12) $$D_{\text{iso}}=S_{t}(\text{TPR},w,L,d)\cdot \frac{k_{s}k_{\text{lin}}}{k_{0}}\cdot \left[\frac{f_{\text{MV}}(d,L)}{F^{0}(\text{TPR},w,L)}\cdot {\text{TMR}}_{w/2}^{w_{\text{iso}}}\right]$$

where $S_{t}(\text{TPR},w,L,d)$ is the transit integral signal in terms of au, obtained by the EPID for the MU, used for a specific beam. This signal is converted by the sensitivity factor, $k_{s}$, in cCU, and corrected by the factors $k_{\text{lin}}$ and $k_{0}$; $F^{0}(\text{TPR},w,L)$ is the ratio $s_{t}^{{}^{\circ}}(\text{TPR},w,L)/D^{0}(\text{TPR},w,L)$, obtained by Eqs. (10) and (11); and $f_{\text{MV}}(d,L)$ are the factors obtained by Eq. (1) averaging the data for the same energy.

All the parameters related to the patient (patient's thickness, isocenter depths, radiological paths, equivalent fields), are easily determined by the physicist on the patient's CT scan containing the isocenter soon after the realization of the treatment plan. Specifically, the parameters, w, w/2, $w_{\text{iso}}$, and d, present in Eq. (12) can be obtained following two steps: i) the patient's CT scan containing the isocenter was used to measure along the beam central axis the patient's geometrical thickness, t, the distance, d, and the isocenter depth, $d_{\text{iso}}$; and ii) calibrated CT numbers were used to determine the mean relative electronic density along the patient's thickness, t, and $d_{\text{iso}}$. In particular, by creating two appropriate regions of interest (ROIs), rectangular in shape 2 mm wide along the patient's geometrical thickness, t, and along the isocenter depth, $d_{\text{iso}}$, the TPS is able to compute the mean relative electronic density values in each of them. Therefore, the water‐equivalent or radiological thicknesses, w, and the depth, $w_{\text{iso}}$, can be determined as the product of t and $d_{\text{iso}}$ by the relative mean physical densities obtained by the linear relation between the electronic density and the physical density.^(^ ^12^ ^)^ The equivalent square field is generally supplied automatically by some TPSs; otherwise, it can be obtained by the Sterling approximation.^(^ ^13^ ^)^ In this pretreatment step, about 10 minutes are needed for the determination of all parameters for a typical plan (i.e., four fields).

An in‐house software, $D_{\text{iso}}$, was developed in MATLAB environment to implement Eq. (12). Specifically, the software reads and converts the EPID images, obtained after the patient's daily treatment, in integral signals s(x,y). Then, the integrated signal on the beam central axis is corrected for $k_{\text{lin}}$, $k_{0}$ and $k_{s}$ for each beam quality index TPR used for patient treatment. The patient's radiological thickness, w, and the beam quality index,TPR, are used in Eqs. (10) and (11) to obtain the generalized midplane doses and the generalized transit signals. These data are then fitted to interpolate the $s^{0}$ and $D^{0}$ for obtaining the ratio $F^{0}$ relative to a specific equivalent field square size, L. For every beam the $D_{\text{iso}}$ value is determined, as well as the ratio R = $D_{\text{iso}}$/$D_{\text{iso},\text{TPS}}$, within few minutes (about one minute for field) after the end of the treatment session.

## III. RESULTS

### A. Factors $f_{\text{MV}}(w,L)$

**Figure 2:**
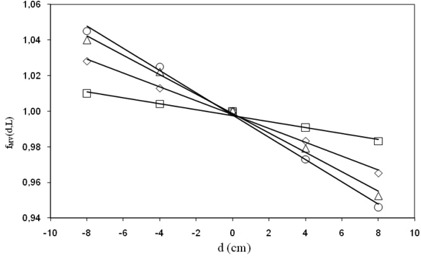
$f_{\text{MV}}(d,L)$ factors obtained for 4×4 (□), 10×10 (⋄), 16×16 (Δ) and $20\times 20\;{\text{cm}}^{2}$ square (O) fields of 15 MV, and the linear fit (continuous lines) by Eq. (13).

The f(TPR d,L) factors (from Eq. (1)) resulted about independent (within 0.2%) of the TPR of the beams with the same MV. Figure 2 shows the $f_{\text{MV}}(d,L)$ average factors obtained for the 15 MV photon beams as a function of the distance, d, for some square fields (L=4,10,16,20 cm). These factors were fitted with linear equations as a function of the distance, d

(13) $$f_{\text{MV}}(d,L)=f_{1}d+1$$

The coefficients $f_{1}$ of the linear fits are reported in Table A1 of Appendix A.

**Table 2:** $k_{0}$ factors (Eq. (4)) obtained for the nine beams of the three linacs examined in this work.

	*Linac A*	*Linac B*	*Linac C*
6 MV	0.660	0.660	0.971
10 MV	0.701	0.701	0.952
15 MV	0.723	0.723	0.943

**Table 3:** $k_{s}$ factors (Eq. (8)) in terms of cCU/au obtained for the nine beams of the three linacs examined in this work.

	*Linac A*	*Linac B*	*Linac C*
6 MV	2.579·$10^{-5}$	2.573·$10^{-5}$	1.655·$10^{-5}$
10 MV	2.840·$10^{-5}$	2.832·$10^{-5}$	2.025·$10^{-5}$
15 MV	2.916·$10^{-5}$	2.908·$10^{-5}$	2.709·$10^{-5}$

**Table 4:** Average values $k_{\text{lin}}$ for different MUs obtained for the three EPIDs used in this work.

	*5 MU*	*20 MU*	*50 MU*	*100 MU*	*200 MU*	*400 MU*	*1000 MU*
$k_{\text{lin}}$	1.016	1.011	1.008	1.000	0.997	0.994	0.990

**Table 5:** Dosimetric and geometrical parameters used for $D_{\text{iso}}$ reconstruction.

*Beam*	*Gantry Angle*	$\rho (g/cm^{3})$	$\;\rho iso(g/cm^{3})$	*z (cm)*	$z_{iso}(cm)$	*w (cm)*	$w_{iso}(cm)$	*L (cm)*	$D_{iso,TPS}(cGy)$	$D_{iso}(cGy)$
1	0°	0.926	0.961	23.4	16.6	21.7	16.0	9.5	128	126.0±3.0
2	130°	0.921	1.050	23.4	10.5	21.6	11.0	9.5	82	82.8±1.5
3	200°	0.947	0.785	25.2	7.2	23.9	5.7	8.8	66	64.1±1.1

Notes: ρ and $\rho_{\text{iso}}$ are the physical density along beam axis across the patient and up to isocenter, respectively; z and $z_{\text{iso}}$ are the geometrical patient thickness and the isocenter depth along beam axis; w and $w_{\text{iso}}$ are the radiological paths along the beam axis; L is the equivalent square field size, $D_{\text{iso},\text{TPS}}$ is the calculated dose by TPS at isocenter and $D_{\text{iso}}$ is the mean measured dose.

**Table A1:** Coefficients $f_{1}$ of the fits performed for the $f_{\text{MV}}(d,L)$ factors as a function of the distance, d, (Eq. (13)) and for the three megavoltage beams.

			*Square Field Size (cm)*			
	*4*	*8*	*10*	*12*	*16*	*20*
6 MV	−7.989E−04	−1.897E−03	−2.642E−03	−3.386E−03	−4.598E−03	−5.933E−03
10 MV	−1.654E−03	−2.553E−03	−3.208E−03	−3.864E−03	−4.316E−03	−5.775E−03
15 MV	−1.673E−03	−3.175E−03	−3.886E−03	−4.598E−03	−5.456E−03	−6.250E−03

**Table A2:** Coefficients $a_{i}(i=1,\ldots ,6)$ of the polynomial surface fits for $D^{0}$ (Eq. (10)) for the six square field sides (L × L ${\text{cm}}^{2}$).

*Coefficient Index*	4×4	8×8	10×10	12×12	16×16	20×20
$a_{1}$	1.35829E+00	1.66047E+00	1.77903E+00	1.56038E+00	1.26118E+00	1.88206E+00
$a_{2}$	−3.89879E−02	−3.67915E−02	−3.51922E−02	−3.55092E−02	−3.38512E−02	−3.27646E−02
$a_{3}$	−1.22685E+00	−2.01130E+00	−2.29958E+00	−1.59924E+00	−6.80369E−01	−2.26615E+00
$a_{4}$	9.90741E−05	5.64815E−05	4.02778E−05	3.14815E−05	1.55093E−05	3.00926E−06
$a_{5}$	1.06570E+00	1.65613E+00	1.84033E+00	1.31302E+00	6.37265E−01	1.65519E+00
$a_{6}$	3.01252E−02	3.02655E−02	2.95331E−02	3.08375E−02	3.02214E−02	3.01669E−02

**Table A3:** Coefficients $b_{i}$ (i = 1,…,10) of the polynomial surface fits for $s_{t}^{0}$ (Eq. (11)) for the six square field sides $\left(L\times L\;{\text{cm}}^{2}\right)$.

*Coefficient Index*	4×4	8×8	10×10	12×12	16×16	20×20
$b_{1}$	1.81339E+02	1.46791E+02	1.80070E+02	1.20870E+02	2.26741E+02	2.03795E+02
$b_{2}$	1.06355E−01	9.49833E−02	5.08104E−02	3.68842E−02	6.71633E−02	2.70425E−02
$b_{3}$	−7.59169E+02	−6.14951E+02	−7.49692E+02	−5.04410E+02	−9.45312E+02	−8.47320E+02
$b_{4}$	1.64812E−03	1.37235E−03	1.72799E−03	1.34544E−03	1.25453E−03	1.56237E−03
$b_{5}$	1.06277E+03	8.62054E+02	1.04396E+03	7.04919E+02	1.31632E+03	1.17763E+03
$b_{6}$	−4.68058E−01	−4.21030E−01	−3.24999E−01	−2.56086E−01	−3.34568E−01	−2.49264E−01
$b_{7}$	−5.63220E−06	−4.14140E−06	−5.83620E−06	−4.38080E−06	−4.31860E−06	−4.32440E−06
$b_{8}$	−4.95105E+02	−4.01812E+02	−4.83628E+02	−3.27226E+02	−6.09431E+02	−5.44300E+02
$b_{9}$	3.73598E−01	3.31875E−01	2.77814E−01	2.12960E−01	2.62765E−01	2.22932E−01
$b_{10}$	−1.30443E−03	−1.05993E−03	−1.37283E−03	−9.94610E−04	−8.75270E−04	−1.32555E−03

In clinical cases, once d (the distance between the isocenter point and the middle patient thickness) and the equivalent square field, L, are both determined for the beam characterized by the MV value, the $f_{\text{MV}}(d,L)$ factor can be determined by interpolation of the data reported in Fig. 2.

### B. Determination of generalized correlation ratios

#### B.1 Generalized midplane doses

Table 2 reports the $k_{0}$ factors determined for the nine photon beams of the three linacs examined in this work.

**Figure 3:**
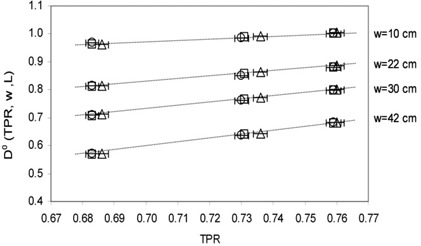
$D^{0}(\text{TPR},w,L)$ values and the linear fits obtained for w=10,22,30 and 42 cm as a function of TPR for the $16\times 16\;{\text{cm}}^{2}$ square field. The symbols refer to linac A (□), linac B (O) and linac C (Δ) in Table 1.

Figure 3 shows the $D^{0}(\text{TPR},w,L)$ values for different w thicknesses as a function of the TPRs for the $16\times 16\;{\text{cm}}^{2}$ square field. The data are reported with the TPR uncertainty (bars of 0.3%). The dose uncertainty was estimated about 2.5% and it is represented by the symbol's size. A good linearity is shown with correlation indexes R≥0.998. The same results were found for the other field dimensions.

**Figure 4:**
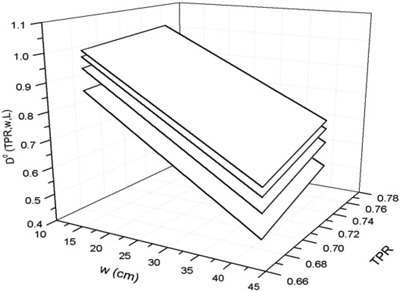
Surfaces obtained by fitting the doses $D^{0}(\text{TPR},w,L)$ as a function of TPR for some square fields 4, 10, 16 and 20 ${\text{cm}}^{2}$.

Figure 4 shows the surfaces fitting the dose $D^{0}(\text{TPR},w,L)$, from Eq. (10), for some square field dimensions used in this paper.

Table A2 in Appendix A reports the six coefficients $a_{i}(i=1,\ldots ,6)$ for the best‐fit of the surface equations.

The residual values (i.e., the differences between the surface and experimental data) were well within ±1%.

#### B.2 Generalized transit signals

The values obtained by the three aSi EPIDs in long‐term periods for the three beam qualities showed a dispersion index of 2% (2 SD), confirming the results obtained in other works^(^ ^14^ ^)^ while, in the short term (during the measurement session), the dispersion was 0.5% (2 SD). Table 3 reports the $k_{s}$ factors obtained for the nine beams of the three linacs used in this work. The observed differences in the values of $k_{s}$ parameter can be explained by considering that $k_{s}$ value depends on two factors: i) the linac dose calibration in terms of cGy/MU, and ii) the manufacturer's calibration of the EPIDs. In this last case, the Elekta Company technicians periodically (every six months) recalibrate the gain image to account for image deterioration due to detector aging and prolonged radiation exposure. This operation modifies the absolute EPID output (i.e., it changes the pixel frame value) and consequently the $k_{s}$ value.

Table 4 reports the average values of the correction factors, $k_{\text{lin}}$, for the three linacs. These factors resulted independent of the beam energies and of the MU/min used within 0.5% (2 SD), in agreement with the findings obtained in other works, for this energy range.^(^ ^5^ ^)^ It must be noted that with respect to the normalization value at 100 MU, the mean EPID response for 1000 MUs was only 1% higher. Therefore, in this dose range, no saturation effect was observed.

With respect to dose rate dependence, a maximum variation of 1.2% was found when changing the dose rate from 50 to 400 MU/min. However, in clinical irradiations, a fixed value of dose rate is chosen (400 MU/min), so this dependence does not constitute a problem.

The amount of ghosting effect for the three aSi EPIDs was evident only for very high number of MUs delivered far from those used in clinical fields; so a ghosting contribution less than 1% was estimated for a number of MUs no greater than 200, and in this work this effect was neglected.

**Figure 5:**
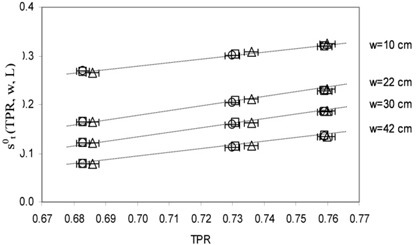
$s_{t}^{0}(\text{TPR},w,L)$ values and the linear fits obtained for w=10,22,30 and 42 cm as a function of TPR for the $16\times 16\;{\text{cm}}^{2}$ square field.

Figure 5 shows the $s_{t}^{0}(\text{TPR},w,L)$ values for different w thickness as a function of the TPRs for the $16\times 16\;{\text{cm}}^{2}$ square field. The data are reported with the TPR uncertainty (bars of 0.3%). The transit signal uncertainty was estimated about 3% and it is represented by the symbol's size. A good linearity is shown, with correlation indexes R≥0.997. The same results were obtained for the other beams.

**Figure 6:**
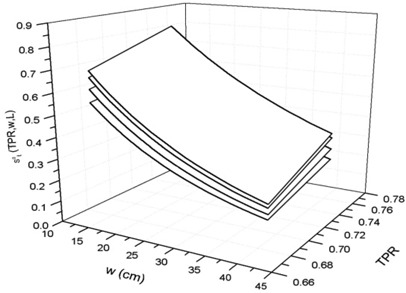
Surfaces obtained by fitting the transit signals $s_{t}^{0}(\text{TPR},w,L)$ as a function of TPRs for the some square fields 4,10, 16 and $20{\text{cm}}^{2}$.

Figure 6 shows the surfaces fitting the $s_{t}^{0}(\text{TPR},w,L)$ data, from Eq. (11), for some square fields used in this paper. Table A3 in Appendix A reports the coefficients $b_{i}(i=1,\ldots ,10)$ for the best‐fit of the surface equations. The residual data between the computed and experimental data were within ±1.5%.

#### B.3 Correlation ratios $F^{0}(\text{TPR},w,L)$ for a clinical case

For each beam of quality index TPR used for patient treatment, a radiological patient's thickness, w, was determined and six generalized midplane doses and the six generalized transit signals, one for every square field size (see Section B.2), were obtained by Eqs. (10) and (11) These data were fitted to obtain by interpolation the data $s_{t}^{0}(\text{TPR},w,L)$ and $D^{0}(\text{TPR},w,L)$ for the patient‐equivalent square field.

### C. Two examples for the $D_{\text{iso}}$ determination

A first example reports the application of Eq. (12) for the $D_{\text{iso}}$ reconstruction in SWEP, using three values of TPR, w and L used in the experimental section. Moreover, the irradiation parameters are:

1. $w_{\text{iso}}=w/2$ – this means d=0 cm and both $f_{\text{MV}}(d,L)=1$ and =1
2. 100 MU delivered with the clinical MU rate. In addition, $k_{\text{lin}}=1$.

Uncertainty factors, ε, can be associated with the $k_{0}$ and $k_{s}$ factors due to the tolerance levels of the beam output factor reproducibility, ${ɛ}_{0}=2\%$ and the EPID signal reproducibility, ${ɛ}_{s}=2\%$.

Thus, Eq. (12) can be rewritten with $k_{s}{ɛ}_{s}$ and $k_{0}{ɛ}_{0}$ for an integral signal, $S_{t}=100\cdot s_{t}(w,L)$

(14) $$D_{\text{iso}}=100\cdot s_{t}(w,L)\frac{k_{s}{å}_{s}}{k_{0}{å}_{0}}\left[\frac{D(w,L)\cdot k_{0}}{s_{t}(w,L)\cdot k_{s}}\right]$$

that is equal to

(15) $$D_{\text{iso}}=100\cdot D(w,L)\left(\frac{{å}_{s}}{{å}_{0}}\right)$$

Of course, the isocenter dose is equal to the midplane dose D(w,L) (cGy/MU) for the specific beam, multiplied for the delivered 100 MU. The propagation of the two uncertainties can supply a global uncertainty of 3%.

**Figure 7:**
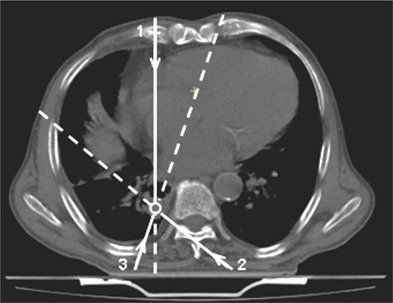
Isocenter CT scans with three beams at 0° (1), 130° (2) and 200° (3) gantry angles. The circle shows the isocenter position.

A second example reports the application of the method in a clinical case. A lung tumor treatment with three conformal fields was shown. The treatment was hypofractionated with a dose fractionation of 300 cGy/fraction in 10 fractions. The CT scans were carried out one week before the beginning of the treatment with three 10 MV x‐ray beams at 0°, 130°, and 200° gantry angles. Figure 7 shows the isocenter CT scan with the three beam gantry angles and the isocenter positioned in the tumor center. The beams central axis up to the isocenter depth are reported as a continuous line. Table 5 reports the dosimetric and geometrical parameters used for $D_{\text{iso}}$ reconstruction. In particular, ρ and $\rho_{\text{iso}}$ are the physical density along beam axis across the patient and until isocenter, respectively; z and $z_{\text{iso}}$ are the geometrical patient thickness and the isocenter depth along beam axis, respectively; w and $w_{\text{iso}}$ are the radiological paths along the beam axis; L is the equivalent square field size and $D_{\text{iso},\text{TPS}}$ is the calculated dose by TPS at isocenter.

**Figure 8:**
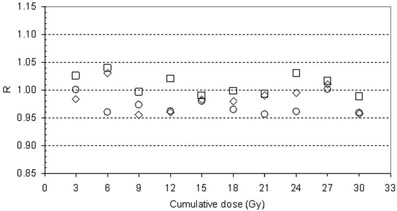
$R=D_{\text{iso}}/D_{\text{iso},\text{TPS}}$ ratios for the beams at 0° (⋄), 130° (O) and 200° (□).

Figure 8 shows the results of the R ratios for the three fields. In all the 10 treatment fractions, the R results were under the action level, so no clinical actions were performed.

## IV. DISCUSSION

In this work, some dosimetric characteristics of three aSi Elekta IviewGT EPIDs, such as the signal reproducibility and the signal linearity with the MU, have been investigated. With regard to short time signal, s, reproducibility was within ±1% (2 SD), while the long term signal reproducibility could be maintained well within ±2% (2 SD). Moreover, the s signal showed a good linearity with the MU, well within ±1% in the range between 20 MU and 400 MU (Table 4). However, it is suggested that one needs to verify the linearity of each EPID in order to evaluate if the $k_{\text{lin}}$ correction factors can be neglected.

A calibration procedure for the aSi‐EPIDs of the Elekta linacs has been reported and for the three beams of 6, 10 and 15 MV, a set of generalized signals $s_{t}^{0}(\text{TPR},w,L)$ have been determined. The generalized correlation ratios $F^{0}(\text{TPR},w,L)$, by Eqs. (10) and (11), together with the set of fMV(d,L) factors, permit one to implement the *in‐vivo* dose reconstruction method in any departments working with Elekta equipment, while avoiding the measurements in solid water‐equivalent phantoms.

An in‐house software has been implemented to supply the isocenter dose $D_{\text{iso}}$ for every beam of the patient once the following are determined: (i) the patient's parameters L, w, $w_{\text{iso}}$, d, and (ii) the linac calibration factors $k_{s}$, $k_{0}$ and $k_{\text{lin}}$.

The TPR values obtained by the three linacs over long‐term periods showed a dispersion in the range of the experimental uncertainty of 0.3%. The linear trends reported in the Figs. 3 and 5 show that the $D^{0}(\text{TPR},w,L)$ and $s_{t}^{0}(\text{TPR},w,L)$ values, can change less than 0.5%, due to the TPR long term variability.

The tolerance level of the method was analyzed in a previous paper,^(^ ^3^ ^)^ but here we briefly report the levels of the principal uncertainties (in terms of 2 SD), estimated for this *in‐vivo* dosimetry method:

(i) ±0.5% for $s_{t}(\text{TPR},w,L)$ due to the signal reproducibility
(ii) ±2.0% for $k_{s}$ due to the daily variations of the EPID sensitivity
(iii) ±0.5% for $k_{\text{lin}}$ due to the accuracy of the data reported in Table 4
(iv) ±2.0% for $k_{0}$ due to the daily machine monitor output variations
(v) ±2.0% for $F^{0}(\text{TPR},w,L)$ due to the accuracy of the fits obtained by Eqs. (10) and (11)
(vi) ±0.7% for f (TPR,d,L) obtained by the fit of Eq. (1)
(vii) ±0.5% for the TMR uncertainty
(viii) ±1.5% due to determination of the water‐equivalent thickness, w, of the patient and the equivalent square field, L
(ix) ±2.0% observed as a consequence of the tolerated variation of ±0.5cm in the beam central axis repositioning during the interfraction patient setup.

Propagating these uncertainties in quadrature, an uncertainty of ±4.4% (2SD) was obtained. Because the results of the proposed *in‐vivo* dosimetry method are reported generally in terms of ratio between the *in‐vivo* reconstructed dose, $D_{\text{iso}}$, and the predicted dose, $D_{\text{iso},\text{TPS}}$ computed by a treatment planning system (TPS), the TPS calculation uncertainty has to be accounted for in the tolerance level determination. The uncertainty in terms of (2 SD) for the $D_{\text{iso},\text{TPS}}$ can be assumed equal to ±2% in homogeneous tissue regions and ±4% in inhomogeneous ones. Propagating in quadrature these last uncertainties with the previous, tolerance levels of ±5% and ±6% can be estimated for the ratios between $D_{\text{iso}}$ and $D_{\text{iso},\text{TPS}}$, in presence of homogenous and inhomogeneous tissues, respectively.

We suggest choosing the tolerance level coincident with the accuracy level according to the philosophy that any deviation larger than the accuracy level must be investigated to determine possible errors in: (i) patient setup, (ii) machine settings, (iii) TPS calculations, and (iv) the patient's morphology changes. These tolerance/action levels seem to be more restrictive than the ones reported by ESTRO^(^ ^1^ ^)^ for the practical method that uses diodes, where for the same treatments, the tolerance/action levels only for the entrance doses have been fixed in that report in 5% and 8% for pelvic and thorax radiotherapy, respectively. As respect to other works^(^ ^15^ ^,^ ^16^ ^)^ that report the same accuracy level for the dose at a reference depth (5 cm), the method here reported supplies the dose in the tumor at the isocenter point that is generally used as the reference point.^(^ ^17^ ^)^

Even if the proposed method is not an independent check of the dosimetry because it requires some of the parameters used by the TPS, it was demonstrated^(^ ^3^ ^)^ that the method is able to check: (i) incorrect patient positioning, (ii) inconsistent CT calibration number, (iii) the patient's morphological modifications. Concerning the morphological tissue changes for lung tumors during the treatment, the method has been used for a dosimetry‐guided radiotherapy that can be well‐integrated with image‐guided radiotherapy.^(^ ^18^ ^)^ Indeed, the change of the transit signals due to the morphological changes of the tumor and the lung tissues that present large different densities can be well detected. As a result, the method was recently also used for a real time control of the $D_{\text{iso},\text{TPS}}$ during the breath‐hold technique adopted to reduce the lung tumor motion.^(^ ^19^ ^)^

## V. CONCLUSIONS

The aim of this work was to implement a generalized model for *in‐vivo* isocenter dose reconstruction by means of Elekta aSi‐EPIDs that uses generalized correlation functions. In particular, in this paper we defined generalized signals, $S^{0}$, and generalized doses, $D^{0}$, by introducing the two parameters, $k_{s}$ and $k_{0}$. This means that in any given facility the clinical physicist, working with Elekta equipment, can use the generalized correlation functions and has only to measure: (a) the beam quality index expressed by ${\text{TPR}}_{20/10}$, (b) the dose at the depth of maximum dose, coincident with the SAD ($D_{\text{SAD}}$) in order to obtain the $k_{0}$ parameter, (c) the EPID transit signal for a $10\times 10\;{\text{cm}}^{2}$ field in terms of au/MU without the phantom on the beam path, in order to obtain the $k_{s}$ parameter. The generalized procedure here reported meets the requirements of efficiency and accuracy, and can be easily included in the quality assurance program of any facility.

Currently, the method is applicable only to open fields containing the central axis. The authors are working on a generalization of the method to include wedged fields and off‐axis dose reconstruction. Moreover, the authors are studying the possibility to obtain the dose reconstruction in real time after the treatment and to extend this generalized procedure also to aSi EPIDs and linacs by other manufacturers.
